# The Role of Immune Checkpoint Molecules for Relapse After Allogeneic Hematopoietic Cell Transplantation

**DOI:** 10.3389/fimmu.2021.634435

**Published:** 2021-03-05

**Authors:** Natalie Köhler, Dietrich Alexander Ruess, Rebecca Kesselring, Robert Zeiser

**Affiliations:** ^1^Department of Medicine I, Medical Center – University of Freiburg, Faculty of Medicine, Albert Ludwigs University (ALU), Freiburg, Germany; ^2^Department of General and Visceral Surgery, Center of Surgery, Medical Center – University of Freiburg, Faculty of Medicine, ALU, Freiburg, Germany

**Keywords:** allogeneic hematopoietic cell transplantation, immune checkpoint, immune checkpoint inhibitor, anti-PD-1, graft-versus-host disease, graft-versus-leukemia

## Abstract

Immune checkpoint molecules represent physiological brakes of the immune system that are essential for the maintenance of immune homeostasis and prevention of autoimmunity. By inhibiting these negative regulators of the immune response, immune checkpoint blockade can increase anti-tumor immunity, but has been primarily successful in solid cancer therapy and Hodgkin lymphoma so far. Allogeneic hematopoietic cell transplantation (allo-HCT) is a well-established cellular immunotherapy option with the potential to cure hematological cancers, but relapse remains a major obstacle. Relapse after allo-HCT is mainly thought to be attributable to loss of the graft-versus-leukemia (GVL) effect and hence escape of tumor cells from the allogeneic immune response. One potential mechanism of immune escape from the GVL effect is the inhibition of allogeneic T cells via engagement of inhibitory receptors on their surface including PD-1, CTLA-4, TIM3, and others. This review provides an overview of current evidence for a role of immune checkpoint molecules for relapse and its treatment after allo-HCT, as well as discussion of the immune mediated side effect graft-vs.-host disease. We discuss the expression of different immune checkpoint molecules on leukemia cells and T cells in patients undergoing allo-HCT. Furthermore, we review mechanistic insights gained from preclinical studies and summarize clinical trials assessing immune checkpoint blockade for relapse after allo-HCT.

## Introduction

Our immune system is an important defense mechanism against invading pathogens as well as against cells that become malignant. Therefore, immunotherapy has become a significant pillar of cancer therapy. The first cellular immunotherapy was established in the 1950s, when Thomas et al. (1) performed the first successful allogeneic hematopoietic cell transplantation (allo-HCT). More recently, blocking physiological control mechanisms of the immune system with immune checkpoint inhibitors (ICI) has led to another major breakthrough in cancer immunotherapy (2). So far, ICI have shown the best clinical responses in patients with solid tumors, while clinical efficacy in most hematological malignancies was lower. However, the possibility to enhance the graft-versus-leukemia (GVL) effect after allo-HCT with ICI has become an enticing concept in the past years. The combination of allo-HCT with ICI is an area of active investigation, which we will discuss in this review.

### Allo-HCT, Graft-versus-Host Disease, and the Graft-versus-Leukemia Effect

Allo-HCT is a potentially curative therapy for diverse benign and high-risk malignant hematological diseases. The most frequent indications for allo-HCT are acute myeloid leukemia (AML), myeloid dysplastic syndromes (MDS), myeloproliferative neoplasms (MPN), and acute lymphocytic leukemia (ALL) (3, 4). An important element for the therapeutic success of allo-HCT is the recognition and elimination of residual malignant cells by allogeneic T cells present in the graft, commonly known as the GVL effect (5). However, the allogeneic donor T cells can also attack healthy tissues of the allo-HCT recipient, most frequently the skin, gastrointestinal tract, and liver. This results in one of the major and potentially lethal complications of allo-HCT, acute graft-versus-host disease (GVHD), which occurs in ca. 30–50% of allo-HCT recipients (6). Furthermore, tumor control by the allogeneic T cells is not extensive and durable enough in all patients. Loss of the GVL effect is thought to be one of the major reasons for relapse of primary disease, which remains the most common cause of death and treatment failure post allo-HCT (3, 7). Therefore, a current major objective is to reinstate the GVL effect without inducing or aggravating GVHD in patients relapsing post allo-HCT. One potential cellular therapy that is currently used to treat relapse after allo-HCT is the infusion of donor lymphocytes (DLI); however, its efficacy and toxicity vary across studies (8, 9). With the clinical breakthrough of immune checkpoint inhibitors (ICI), boosting the GVL effect with ICI post allo-HCT became a tempting concept.

### ICI and Immune Related Adverse Events

**Figure 1:**
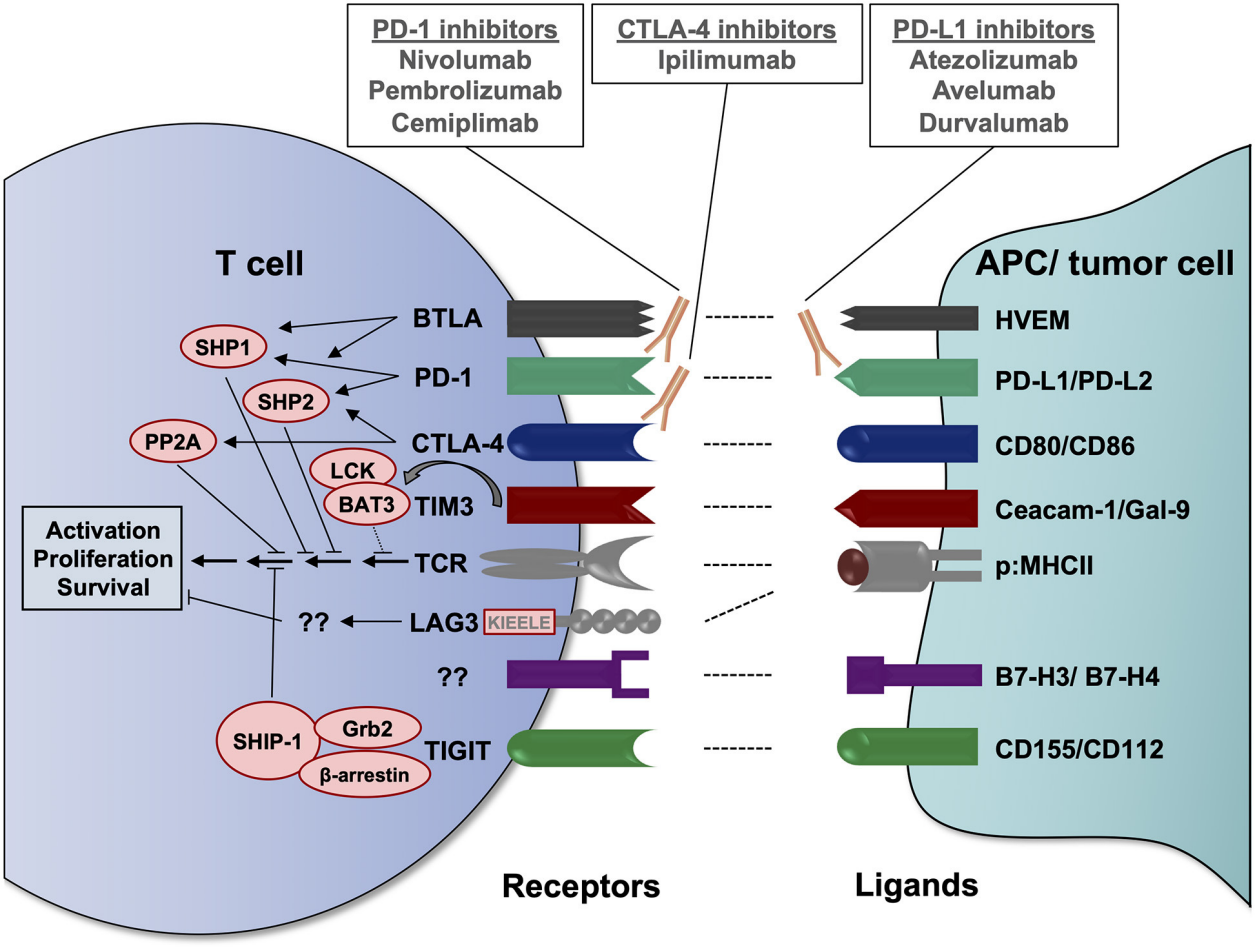
Important inhibitory immunoreceptors (immune checkpoints), their ligands and known signaling mechanisms. Immune checkpoints expressed on the T cell surface bind to their respective ligands on antigen-presenting cells (APC) or tumor cells, resulting in an inhibitory signal to the T cell. These interactions may occur in lymph nodes at the initiation of T cell responses or in peripheral tissues/tumor sites during the T cell effector response. Via its immunoreceptor tyrosine-based inhibitory motif (ITIM) and its immunoreceptor tyrosine-based switch motif (ITSM), PD-1 can recruit the SH2 domain-containing tyrosine phosphatases SHP1 and SHP2, which dephosphorylate TCR signaling mediators, including ZAP70 and Lck, as well as CD28 signaling mediators (12, 13). CTLA-4 competes with CD28 for the binding of B7 molecules, preventing the CD28 co-stimulatory signal, and recruits phosphatases that counteract T cell activation, including SHP2 and protein phosphatase 2A (PP2A) (12, 14). Similar to PD-1, BTLA can recruit the phosphatases SHP1 and SHP2 to inhibit TCR and CD28 downstream signaling (15, 16). Upon T cell activation and in the absence of TIM3 ligands, HLA-B-associated transcript 3 (BAT3) is bound to the cytoplasmic domain of TIM3 and to the tyrosine kinase LCK, which is involved in proximal TCR signaling. Upon binding of its ligands Ceacam-1 or gal-9, tyrosine residues in the intracellular domain of TIM3 are phosphorylated, causing the release of BAT3 from its cytoplasmic tail and recruitment of tyrosine phosphatases, contributing to TCR signaling inhibition (17–19). LAG3 does not contain an ITIM or ITSM inhibitory motif, but different well-conserved amino acid motifs, including a KIEELE motif, which potentially contribute to its T cell inhibitory function (20, 21). The exaxt signaling mechanisms remain to be elucidated. Upon binding of its ligands CD155 (PVR) or CD112 (PVRL2), TIGIT has been proposed to recruit the adapter proteins Grb2 and β-arrestin and the SH2-containing inositol phosphatase-1 (SHIP-1), which interferes with phosphoinositide 3-kinase (PI3K), mitogen-activated protein kinase (MAPK) and NF-κB signaling (22, 23). However, these findings have been made in NK cells and have yet to be confirmed in the T cell setting. BAT3, HLA-B-associated transcript 3; BTLA, B and T lymphocyte attenuator; Ceacam-1, carcinoembyronic antigen-related cell adhesion molecule-1; CTLA-4, cytotoxic T-lymphocyte-associated protein 4; Gal-9, galectin 9; Grb2, growth factor receptor-bound protein 2; HVEM, herpesvirus entry mediator; LAG3, lymphocyte activation gene-3; p:MHCII, peptide:major histocompatibility complex II; PD-1, programmed cell death protein 1; PD-L, PD-1 ligand; PP2A, protein phosphatase 2A; PVRL2, poliovirus receptor-related 2; SHIP-1, SH2-containing inositol phosphatase-1; SHP, SH2 domain-containing tyrosine phosphatase; TCR, T cell receptor; TIGIT, T cell immunoreceptor with Ig and ITIM domains; TIM3, T cell immunoglobulin and mucin-domain containing protein 3.

Immune checkpoints are physiological control mechanisms of our immune system, which are crucial for maintaining immune homeostasis and the prevention of autoimmune reactions (10). As a general concept, inhibitory immunoreceptors expressed on the surface of T cells interact with specific ligands leading to reduced T cell activation and/or T cell apoptosis. The inhibitory checkpoint ligands can be expressed on stromal cells or antigen-presenting cells (APC) but also on tumor cells, which exploit these regulatory mechanisms to escape the anti-tumor immune response (11). In recent years, various different inhibitory immuno-receptors, also known as immune checkpoints, have been identified and analyzed for their role in cancer, including but not limited to PD-1, CTLA-4, LAG3, TIM3, TIGIT, and BTLA (summarized in Figure 1).

Inhibition of the two best described immune checkpoints, CTLA-4 and PD-1, using monoclonal antibodies has led to a breakthrough in cancer immunotherapy in the recent decade, showing remarkable responses and improved overall survival (OS) in many different solid tumors (24–27). Blocking the interaction of CTLA-4 and its ligands from the B7 family (CD80/CD86) using antibodies had shown first promising anti-tumor effects in murine cancer models in 1996 (28). Similarly, early studies demonstrated that interaction of PD-1 and its ligand PD-L1 on tumor cells represents a tumor immune escape mechanism and that blockade of the PD-1/PD-L1 axis reduced tumor growth in experimental models (29). These reports set the cornerstone for today's rapid clinical successes in the field of immune checkpoint blockade. To date, multiple immune checkpoint inhibitors (blocking either CTLA-4, PD-1, or PD-L1) are approved for more than 15 different cancer entities, however, efficacy has so far been most promising in solid tumors (Table 1). By systemically increasing T cell activity, ICI can also enhance autoimmune responses and induce inflammatory side effects, which are termed immune-related adverse events (irAEs) (30). These are more common and severe with CTLA-4 blockade than with PD-1/PD-L1 blockade and can principally affect any organ system (30–32). IrAEs can be life-threatening, but are usually well manageable with steroid treatment (31). Nevertheless, both irAEs and GVHD are complications that require close monitoring when combining ICI with allo-HCT and will be topics covered in this review.

**Table 1:** Currently approved immune checkpoint inhibitors (ICI) for cancer immunotherapy.

**Name of ICI**	**Target**	**FDA-approved indications**	**FDA-approved indications**
		**Solid tumors**	**Hematological malignancies**
Ipilimumab (Yervoy®)	CTLA-4	•Melanoma	
Nivolumab (Opdivo®)	PD-1	•Melanoma •NSCLC •SCLC •Renal cell carcinoma •Squamous cell carcinoma of the head and neck •Urothelial carcinoma •Hepatocellular carcinoma •Esophageal squamous cell carcinoma	•Classical HL
Pembrolizumab (Keytruda®)	PD-1	•Melanoma •NSCLC •SCLC •Renal cell carcinoma •Head and neck squamous cell cancer •Urothelial carcinoma •Gastric cancer •Esophageal cancer •Cervical cancer •Endometrial carcinoma •Hepatocellular carcinoma •Merkel cell carcinoma •Microsatellite Instability-High (MSI-H) or mismatch repair deficient cancer^*^ •Tumor Mutational Burden-High^*^ (TMB-H) cancer •Cutaneous squamous cell carcinoma	•Classical HL •Primary mediastinal large B cell lymphoma
Cemiplimab (Libtayo®)	PD-1	•Cutaneous squamous cell carcinoma	
Ipilimumab + Nivolumab	CTLA-4 + PD-1	•Melanoma •Renal cell carcinoma •Metastatic colorectal cancer •Hepatocellular carcinoma •NSCLC •Malignant pleural mesothelioma	
Atezolizumab (Tecentriq®)	PD-L1	•Melanoma •Urothelial carcinoma •NSCLC •SCLC •Triple-negative breast cancer •Hepatocellular carcinoma	
Avelumab (Bavencio®)	PD-L1	•Urothelial carcinoma •Renal cell carcinoma •Merkel cell carcinoma	
Durvalumab (Imfinzi®)	PD-L1	•Urothelial carcinoma •NSCLC •SCLC	

*CTLA-4, cytotoxic T-lymphocyte-associated protein 4; HL, Hodgkin lymphoma; ICI, immune checkpoint inhibitor; NSCLC, non-small lung cancer; PD-1, programmed cell death protein 1; PD-L1, PD-1 ligand 1; SCLC, small cell lung cancer*.

* *Limitation: The safety and effectiveness of pembrolizumab in pediatric patients with MSI-H/TMB-H central nervous system cancers have not been established*.

## Immune Checkpoints and Relapse After ALLO-HCT

### Expression of Immune Checkpoint Ligands on Leukemia Cells in Patients Undergoing allo-HCT

Relapse after allo-HCT is thought to be attributed mainly to the loss of the GVL effect and hence the escape of tumor cells from the allogeneic immune response. Various different mechanisms of immune escape from the GVL effect post allo-HCT exist, which have recently been reviewed (33). These include downregulation of MHC molecules, production of anti-inflammatory factors and metabolically active enzymes, loss of pro-inflammatory cytokine production, and notably the expression of immune checkpoint ligands (33). Upregulation of immune regulatory molecules on AML blasts has been shown to be a distinctive characteristic and driver of AML relapse post allo-HCT (34). Already in 2011, a study focusing on the PD-1/PD-L1 axis reported increased PD-L1 expression on myeloid leukemia cells after IFNγ and TNFα stimulation as well as PD-1 expression on minor histocompatibility antigen (MiHA)-specific memory CD8 T cells (35). Subsequently, comprehensive immuno-phenotyping of AML blasts before and after allo-HCT revealed an upregulation of PD-L1, B7-H3, poliovirus receptor-related 2 (PVRL2/CD112, ligand for TIGIT) and CD80 at relapse after allo-HCT compared to initial diagnosis (34). Concomitantly, the percentage of PD-1 expressing T cells was higher at post-transplantation relapse than in healthy controls and in AML patients before allo-HCT. To investigate the functional relevance of these findings, the authors used co-culture experiments of leukemia blasts and donor-derived T cells from one patient. *Ex vivo* addition of anti-PD-L1 blocking antibody caused increased T cell proliferation and IFNγ production, indicating that in some patients with deregulated PD-1/PD-L1 expression, checkpoint inhibition might reinstate the GVL effect against relapsed AML (34).

### Expression of Inhibitory Checkpoint Receptors on T Cells in Patients Undergoing allo-HCT

An increasing number of studies report on the co-expression of inhibitory checkpoint receptors on donor T cells and their correlation with relapse post allo-HCT. Jain and colleagues found that PD-1 expression was elevated both on peripheral blood (PB) T cells from relapsed as well as non-relapsed patients having undergone human leukocyte antigen (HLA)-matched stem cell transplantation. This indicates that PD-1 is not the sole predominant marker for leukemia-specific T cell exhaustion in patients relapsing after allo-HCT (36). Deeper analyses using single-cell RNA sequencing of one patient sample revealed that LAG3 and TIM3 were overexpressed in leukemia antigen-specific T cells (36). In line with these data, the frequency of peripheral blood PD-1-high TIM3^+^ T cells was strongly associated with leukemia relapse in 11 AML patients who received allo-HCT (37). Importantly, the PD-1-high TIM3^+^ cells showed functional signs of exhaustion, including reduced production of IL-2, IFNγ and TNFα, and their increase occurred before clinical diagnosis of leukemia relapse, suggesting their predictive value (37). Similarly, Williams et al. (38) reported a trend toward a higher frequency of CD8^+^PD-1^+^TIM3^+^ T cells and CD8^+^PD-1^+^LAG3^+^ T cells in the bone marrow (BM) of AML patients with relapse. These findings were confirmed in a study involving 32 AML patients relapsing or maintaining complete remission (CR) after allo-HCT (39). In the BM of relapsing patients, a higher proportion of CD8^+^ T cells expressed CTLA-4, PD-1 and TIM3 when compared to CR patients (39). This was only the case in patients who underwent HLA-identical allo-HCT, while the profile of inhibitory receptors did not correlate with clinical outcome after haploidentical transplantation, hypothetically due to the higher degree of HLA-mismatch and therefore and increased inflammatory cytokine milieu. Of note, the inhibitory receptor expressing T cells displayed a skewed T cell receptor (TCR) repertoire at relapse and better recognized and eliminated matched leukemic blasts *in vitro* when compared to inhibitory receptor negative T cells, indicating that inhibitory receptor expression marks leukemia-specific T cells (39). In agreement with this hypothesis, PD-1, TIGIT, and KLRG-1 were highly co-expressed on circulating MiHA-reactive CD8 T cells after allo-HCT and this expression was associated with relapse risk (40). A further study by Hattori et al. (41) confirmed that a high expression of TIGIT in BM samples of AML patients after allo-HCT correlated with poor overall survival (OS) and progression-free survival (PFS) as well as decreased incidence of acute GVHD, indicating a regulatory effect of TIGIT on allo-reactive cells.

In addition to its expression on exhausted T cells, TIM3 is a marker for acute myeloid leukemia stem cells (LSCs), which discriminates these cells from normal hematopoietic stem cells (42, 43). In a cohort of 57 AML patients treated with allo-HCT, high percentages of TIM3^+^ LSC at engraftment were a significant independent risk factor for relapse after allo-HCT (44).

### Preclinical ICI Animal Studies

#### CTLA-4

The T cell surface molecules CD28 and CTLA-4 are structurally related and both molecules bind to B7-1 (CD80) and B7-2 (CD86), transmitting T cell stimulatory and inhibitory downstream signals, respectively (45). An early study by Blazar et al. (46) showed that blockade of CTLA-4 at an early time point during allo-HCT augmented alloreactivity, resulting in accelerated GVHD lethality in a major histocompatibility complex (MHC) mismatched mouse model of bone marrow transplantation (BMT). In contrast, treatment with anti-CTLA-4 mAb at a later time point post-BMT in the context of DLI strongly enhanced the GVL effect, while only mildly increasing GVHD (46). Delayed CTLA-4 blockade induced a host-derived anti-leukemic effect in a MiHA-mismatched BMT mouse model, while not inducing GVHD, but an autoimmune syndrome with autoimmune hepatitis and circulating anti-DNA auto-antibodies (47). Importantly, both the anti-leukemic effect and the autoimmune pathology were mediated by host and not donor T cells, but depended on the allogeneic component, as neither effect was seen after syngeneic BMT (47).

#### PD-1/PD-L1/2 Axis

Numerous studies have addressed the question of how PD-1 and its ligands PD-L1 and PD-L2 regulate the delicate balance between GVHD and GVL post-allo-HCT. Already in 2003, Blazar et al. (48) demonstrated in murine models that blocking either PD-1 or PD-L1 aggravates GVHD in an IFNγ dependent mechanism (48). Blocking both CTLA-4 and PD-1 together was additive in enhancing GVHD, indicating the non-redundancy of these pathways. In a follow-up study, they identified the PD-1/PD-L1 axis to be predominant in regulating GVHD development, as compared to PD-1/PD-L2 interaction, and that PD-L1 expression on host parenchymal cells is critical for the suppression of acute GVHD (49). However, the effects of PD-1/PD-L1/PD-L2 blockade on the GVL response were not assessed in these studies.

Asakura and colleagues demonstrated that blocking PD-L1 antibody treatment early after allo-HCT improved T cell effector functions and GVL activity in mice, but this occurred at the expense of aggravated GVHD (50). In contrast, *in vivo* PD-L1 blockade at later time points after DLI (day 48–60) was able to enhance cytotoxic T lymphocyte (CTL) activity and GVL effects without induction of GVHD (51). Similarly, the efficacy of adoptive transfer of gene-modified leukemia-specific T cells late (56 days) after T cell-depleted BM transplantation, could be enhanced by additional systemic blockade of PD-L1, without inducing GVHD (52). Michonneau et al. (53) identified the differentiation of GVHD and GVL responses by anatomical differences in CTL activity and PD-L1/PD-L2 expression in a mouse model of single MiHA-mismatched allo-HCT. PD-1 ligand expression was low on liver antigen-presenting cells (APCs) and high on APCs and endothelial cells in the lymph nodes, resulting in GVHD development and local tumor immune escape, respectively. PD-1 blockade was able to restore CTL killing activity in lymph nodes, together indicating that the PD-1 pathway is not equally engaged in all organs (53). Further work by the Blazar group revealed that, in contrast to host PD-L1 expression, PD-L1 expression on donor T cells augments GVHD in murine allo-HCT models (54). *Pdl1* deficient donor T cells caused reduced GVHD, while they importantly still displayed potent GVL function, suggesting that selective inhibition of PD-L1 on donor T cells might ameliorate GVHD, while preserving the GVL effect (54). Taken together, these studies indicate a time-, organ- and cell type-dependent function of the PD-1/PD-L1/PD-L2 axis during allo-HCT.

A recent study assessed the mechanisms of GVL failure using an elegant mouse model, in which GVL is exclusively mediated by alloreactive CD8^+^ T cells recognizing the MiHA H60, making it possible to specifically track and analyze the GVL-inducing T cells (55, 56). Next to insufficient H60 presentation, the GVL effect in this model failed due to the development of leukemia-specific T cell exhaustion, characterized by expression of the inhibitory receptors PD-1, TIGIT, LAG3, and TIM3 and the transcription factor TOX, which has recently been shown to drive T cell exhaustion (57–61). Blockade of PD-1 was able to reverse the T cell exhaustion phenotype and restore the GVL effect, whereas blockade of TIM3, LAG3, and TIGIT were not, suggesting that PD-1 may be the dominant inhibitory checkpoint contributing to GVL failure in mice (55).

### Clinical Evidence

Translating the above-described preclinical evidence into clinical application of ICI for patients relapsing after allo-HCT has been challenging, due to understandable concern regarding the occurrence of immune-related side effects, in particular severe GVHD. To date there is only limited data regarding the efficacy of checkpoint inhibitors before or after allo-HCT in hematological malignancies other than Hodgkin lymphoma (HL). In the following paragraphs, we focus on clinical trials that have assessed CTLA-4 or PD-1 blockade in patients relapsing after allo-HCT. Major studies evaluating ICI therapy in hematological malignancies relapsing after allo-HCT are summarized in Table 2.

**Table 2:** Selected clinical trials of checkpoint inhibitor therapy in hematological malignancies following allo-HCT.

**References**	**Intervention**	**Study population**	**Study type**	**Outcome**
Herbaux et al. (62)	Nivolumab (q2w, 3 mg/kg)	HL relapsed after allo-HCT (*n* = 20)	Retrospective study	ORR/CR/PR = 95/42/52% 12 month PFS/OS = 58.2/78.7%
Haverkos et al. (63)	Nivolumab (q2w, 3 mg/kg): *n* = 28 Pembrolizumab (q3w, 200 mg): *n* = 3	Lymphoma relapsed after allo-HCT (*n* = 31) HL: *n* = 29; FL + HL: *n* = 1; transformed FL: *n* = 1	Retrospective study	ORR/CR/PR = 77/50/27% Median PFS/OS = 19 months/not reached
Davids et al. (64, 65)	Ipilimumab (q3w) 3 mg/kg: *n* = 6 5 mg/kg: *n* = 15 10 mg/kg: *n* = 22	Hematological malignancies relapsed after allo-HCT (*n* = 43) AML: *n* = 18; HL: *n* = 7; NHL: *n* = 5; CLL: *n* = 3; MM: *n* = 3; MDS: *n* = 3; ALL: *n* = 2; MPN: *n* = 1; CMML: *n* = 1	Phase I/Ib	3 mg/kg: no response 5 mg/kg: ORR/CR/PR = 23/0/23% median PFS/OS = 3.4/7 months 10 mg/kg: ORR/CR/PR = 32/23/9% median PFS/OS = 9.4/28.3 months
Khouri et al. (66)	Lenalidomide (10 mg/day for 21 days) + Ipilimumab (3 mg/kg, single dose) Repeated for 2 cycles	Lymphoid malignancies relapsed after allo-HCT (*n* = 19) MCL: *n* = 3; CLL: *n* = 2; FL: *n* = 2; THL: *n* = 1; DLBCL: *n* = 1; ALCL: *n* = 1	Phase II	ORR/CR/PR = 70/40/30% 90% OS at median follow-up of 20.5 months
Holderried et al. (67)	Ipilimumab (*n* = 10) Nivolumab (*n* = 5) Nivolumab + DLI (*n* = 5) Nivolumab + Ipilimumab (*n* = 1)	Hematological malignancies relapsed after allo-HCT (*n* = 21) MDS/AML: *n* = 12; NHL: *n* = 5; ALL: *n* = 2; MF: *n* = 2	Retrospective study	Overall ORR/CR/PR = 43/14/29% Ipilimumab: ORR = 20% Nivolumab: ORR = 40% Nivolumab + DLI: ORR = 80% Overall median OS = 79 days Ipilimumab: median OS = 39 days Nivolumab (±DLI): median OS = 282 days
Kline et al. (68)	Pembrolizumab (q3w, 200 mg)	Hematological malignancies relapsed after allo-HCT Interim analysis (*n* = 11) AML: *n* = 8; DLBCL: *n* = 2; HL: *n* = 1 Planned *n* = 26	Phase I	ORR/CR/PR = 29/29/0% (CR reached in 1 DLBCL and 1 HL patient)
Davids et al. (69)	Nivolumab (q2w) 1 mg/kg: *n* = 6 0.5 mg/kg: *n* = 22	Hematological malignancies relapsed after allo-HCT (*n* = 28) AML: *n* = 10; MDS: *n* = 7; HL: *n* = 5; NHL: *n* = 3; CLL: *n* = 1; CMML: *n* = 1; Leukemia NOS: *n* = 1	Phase I/Ib	1 mg/kg: ORR/CR/PR = 50/17/33% 0.5 mg/kg: ORR/CR/PR = 23/0/23% median PFS/OS = 3.7/21.4 months

*ALCL, anaplastic large T-cell lymphoma; allo-HCT, allogeneic hematopoietic cell transplantation; ALL, acute lymphoblastic leukemia; AML, acute myeloid leukemia; CLL, chronic lymphocytic leukemia; CR, complete remission; DLBCL, diffuse large B-cell lymphoma; DLI, donor lymphocyte infusion; FL, follicular lymphoma; HL, Hodgkin lymphoma; MCL, mantle cell lymphoma; MDS, myelodysplastic syndrome; MF, myelofibosis; MM, multiple myeloma; MPN, myeloproliferative neoplasm; NHL, non-Hodgkin lymphoma; NOS, not otherwise specified; ORR, overall response rate; OS, overall survival; PFS, progression-free survival; PR, partial remission; THL, triple-hit lymphoma*.

#### CTLA-4 Blockade Post allo-HCT

An early dose escalation trial by Bashey et al. (70) demonstrated an acceptable safety profile of ipilimumab in 29 patients with malignancies that were recurrent or progressive after allo-HCT. The underlying disease of the majority of patients was HL (48%) or multiple myeloma (21%). A single infusion of ipilimumab at doses from 0.1 up to 3 mg/kg did not result in acute or chronic GVHD induction, while four patients developed irAEs. However, it has to be noted that patients with prior grade 3 or 4 acute GVHD development were excluded from this study. Three patients with lymphoid malignancies demonstrated objective disease responses after a single dose of 1 or 3 mg/kg ipilimumab (70). In a proportion of the patients, increases in activated CD4^+^ T cells were observed after ipilimumab infusion (71).

A subsequent phase I/Ib study analyzed safety and efficacy of ipilimumab in 28 patients with hematological malignancies relapsing after allo-HCT with no history of prior grade 3 or 4 acute GVHD (64). Ipilimumab dosage was 3 or 10 mg/kg every 3 weeks for a total of 4 doses, with additional doses every 12 weeks for up to 60 weeks in patients with clinical benefit. Response to treatment was dose-dependent, with no response observed in patients who received a dose of 3 mg/kg, while in the 10 mg/kg cohort (*n* = 22) 23% of patients achieved a CR and 9% a PR. GVHD that led to treatment discontinuation, but was responsive to glucocorticoids, occurred in 4 patients, and irAEs, including one fatality, were observed in 6 patients. At a median follow-up of 27 months, OS and PFS were 54 and 32% for the 10 mg/kg group, respectively (64). An update of this study reported about an intermediate dose (5 mg/kg) phase Ib extension cohort including 15 additional patients (65). At 5 mg/kg ipilimumab, partial responses were also observed, but the reduced dose did not improve the rate of GVHD or irAEs (65).

Furthermore, combination treatment of lenalidomide (10 mg/day for 21 days) followed by ipilimumab (3 mg/kg) in ten patients relapsing after allo-HCT has been assessed in a phase II trial (66). One patient with known GVHD history had a flare of his symptoms after the first lenalidomide cycle that precluded further treatment, while all others completed treatment without GVHD development. Overall response rate (ORR) was 70% (4 CR, 3 PR) and at a median follow-up time of 20.5 months 90% of patients were alive. Importantly, ipilimumab plus lenalidomide combination treatment led to significantly increased numbers of circulating CD4^+^ICOS^+^FoxP3^−^ conventional T cells (66).

#### PD-1 Blockade Post allo-HCT

##### Hodgkin Lymphoma

Given the clinical success of anti-PD-1 therapy in HL, multiple early case reports and case series describing the use of anti-PD-1 antibodies in patients with HL relapsing after allo-HCT have been published. In these reports, some patients benefitted from anti-PD-1 therapy post allo-HCT without the occurrence of serious side effects [nivolumab (72–74) and pembrolizumab (75)], while other patients developed severe toxicity with fatalities from GVHD [nivolumab (76) and pembrolizumab (77, 78)].

Herbaux et al. (62) retrospectively assessed the efficacy and toxicity of nivolumab in 20 HL patients relapsing after allo-HCT. Response rates were high (ORR 95%, CR 42%, PR 52%) and 1-year PFS and OS were 58.2 and 78.7%, respectively. Acute GVHD occurred in six patients (30%) within 1 week after the first nivolumab dose and was manageable with standard GVHD treatment. All six patients had prior history of acute GVHD. Time between allo-HCT and nivolumab treatment start was significantly shorter in patients developing GVHD (62). Another retrospective study by Haverkos et al. (63) revealed promising response rates but also high GVHD frequency after anti–PD-1 treatment. Thirty one patients with lymphoma relapse after allo-HCT were treated with nivolumab (*n* = 28) or pembrolizumab (*n* = 3), resulting in an ORR of 77% (15 CR, 8 PR). However, 55% of patients developed GVHD already after 1–2 doses of anti-PD-1 treatment, including grade III-IV GVHD in 9 patients and 8 deaths related to treatment-emergent GVHD (4 acute GVHD and 4 chronic GVHD) (63).

##### Hematological Malignancies Other Than HL

While the above-described studies mainly included patients with HL, there is increasing interest in the possibility to use checkpoint blockade in the context of myeloid malignancies relapsing post allo-HCT. In a retrospective multi-center study, 21 patients with malignancies other than HL (*n* = 12 MDS/AML, *n* = 5 NHL, *n* = 2 ALL, *n* = 2 myelofibrosis) relapsing after allo-HCT were treated with ICI (67). Patients received either nivolumab or ipilimumab alone, a combination of both, or a combination of nivolumab with DLI. The ORR was 43% (3 CR, 6 PR), with higher response rates observed in patients receiving nivolumab plus DLI (ORR = 80%) compared to patients receiving nivolumab alone (ORR = 40%) or ipilimumab alone (ORR = 20%). However, grade III/IV aGvHD or moderate/severe cGvHD developed in 29% of patients, of which 83% were steroid-refractory (67). Kline et al. (68) presented early results from a still recruiting phase I study of pembrolizumab for the treatment of AML, MDS or B cell lymphoma relapse following allo-HCT. However, in 8 patients with AML treated so far, pembrolizumab seemed to have only limited effect with a best response of SD observed in 2 patients (68).

Recently, data from the first prospective trial of nivolumab for relapsed hematological malignancies (myeloid *n* = 19, lymphoid *n* = 9) after allo-HCT were reported (69). Nivolumab was administered every 2 weeks starting with a 1 mg/kg cohort (*n* = 6), of which two experienced dose-limiting toxicity from irAEs, resulting in dose reduction to 0.5 mg/kg for the remaining 22 patients. Anti-tumor activity was only modest, with an ORR of 29% and 1-year PFS and OS of 23 and 56%, respectively. ORR was higher in patients with lymphoid malignancies (44%) as compared to patients with myeloid malignancies (21%). Chronic or acute GVHD occurred in 39% of patients and was fatal in two patients (69).

In a recent study, low-dose regimens of pembrolizumab and nivolumab in the post allo-HCT settings were tested in a small patient cohort. Two heavily pretreated patients with HL relapsing after allo-HCT received 40 mg of nivolumab every 2 weeks (79). One of them remained in CR at 22 months; the other remained in PR at 6 months at the time point of analysis. Both patients did not develop any irAEs (79). In contrast, another recent phase I study of low-dose nivolumab as maintenance therapy post allo-HCT reported on unexpected severe toxicities (80). Four patients with AML or MDS were treated with nivolumab at 1 mg/kg every 2 weeks for four doses. All of them developed irAEs, and two patients experienced serious adverse events, including grade 4 neutropenia and grade 3 autoimmune encephalopathy, resulting in study termination (80).

Taken as a whole, these studies indicate that lower doses of anti-PD-1 treatment might have the potential to induce responses without inducing severe immunological complications, but also highlight the need for further dose-finding studies, potentially resulting in differing optimal dosing regimens for different underlying malignancies. Overall, the studies so far suggest that frequency and severity of immune-related adverse events and GVHD are higher in anti-PD-1 treated patients than in anti-CTLA-4 treated patients in the post allo-HCT setting.

#### Ongoing Clinical Trials

Multiple phase I and phase II clinical trials of checkpoint inhibitor therapy following allo-HCT are currently ongoing (summarized in Table 3). Many of them focus not only on HL but on AML and MDS and both ICI monotherapy and combination therapies are studied. The results of these trials could give more insight into efficacy and safety of ICI in the post-transplantation settings in diseases other than HL and the results are eagerly anticipated.

**Table 3:** Selected ongoing clinical trials of checkpoint inhibitor therapy in hematological malignancies following allo-HCT.

**Clinical trial identifier**	**Intervention**	**Study population**	**Phase**	**Planned n**	**Study start**	**Status**
**ICI monotherapy**						
NCT03146468	Nivolumab	Relapsed/residual hematological malignancies after allo-HCT	II	14	May 2017	Active, not recruiting
NCT02981914 (68)	Pembrolizumab	AML/MDS/B cell lymphoma relapsed after allo-HCT	I	26	Mar 2017	Recruiting
NCT03286114	Pembrolizumab	AML/ALL/MDS relapsed after allo-HCT	I/Ib	20	December 2017	Recruiting
2017-002194-18 (EudraCT)	Nivolumab	Relapse of AML after allo-HCT	I/II	20	March 2018	Active, not recruiting
NCT04361058	Nivolumab	High risk AML/MDS relapsed afterarm A: HLA-matched unrelated donor allo-HCTarm B: HLA-haploidentical allo-HCT	I	36	April 2020	Recruiting
**ICI combination therapy**						
NCT02846376	Nivolumab vs. Ipilimumab vs. Nivolumab + Ipilimumab	AML/MDS at risk for relapse after allo-HCT	I	8	March 2019	Active, not recruiting
NCT03600155	Nivolumab vs. Ipilimumab vs. Nivolumab + Ipilimumab	AML/MDS relapsed/refractory after allo-HCT	Ib	55	October 2018	Recruiting
NCT04128020	Nivolumab + Azacitidine	AML/high risk MDS after reduced-intensity allo-HCT	I	48	October 2019	Recruiting

*allo-HCT, allogeneic hematopoietic cell transplantation; ALL, acute lymphoblastic leukemia; AML, acute myeloid leukemia; HLA, human leukocyte antigen; ICI, immune checkpoint inhibitor; MDS, myelodysplastic syndrome*.

## Discussion and Future Perspectives

Allo-HCT is a well-established cellular immunotherapy option with the potential to cure high-risk hematological malignancies. However, relapse remains the major cause of death and treatment failure after allo-HCT. By inhibiting negative regulators of the immune response, checkpoint blockade can increase anti-tumor immunity, but has been primarily successful in solid cancer therapy so far.

On the one hand, boosting the allogeneic immune response post allo-HCT by blocking immune checkpoints is an appealing concept to prevent or treat relapse of hematological cancers. Numerous studies have found a connection between the expression of inhibitory checkpoints and disease relapse post allo-HCT. Clinical trials indicate therapeutic potential for the combination of these two immunotherapies, although lymphoid malignancies seem to be more responsive than myeloid malignancies thus far. Future preclinical studies and clinical trials will be crucial to further assess which checkpoints are the best therapeutic targets, taking into consideration the underlying disease, risk of side effects, optimal dose, timing, and therapy duration. The results of ongoing studies focusing on myeloid malignancies and assessing dual checkpoint blockade post allo-HCT are eagerly awaited to answer these open questions. Furthermore, the increased expression of other immune checkpoints on T cells in murine GVL models and in patients relapsing after allo-HCT, including TIM3 and TIGIT, suggests that novel immune checkpoint inhibitors blocking these molecules might offer potential treatment options post allo-HCT.

On the other hand, both allo-HCT and ICI therapy commonly induce inflammatory side effects, referred to as GVHD and irAEs, respectively. Although the roots and pathogenesis of these complications are distinct (allo- vs. auto-immunity), some patho-mechanisms seem to be shared between them, potentially adding up if these therapies are combined. For example, we and others found that the microRNA miR-146a is involved in the regulation of both acute GVHD after allo-HCT and irAEs of ICI therapy (81–84), indicating shared regulatory pathways in these complications. Therefore, the monitoring of immunological complications is of high importance for patients treated with ICIs before or after allo-HCT. Potential strategies to prevent or manage GVHD and irAEs in the context of ICI include starting ICI treatment at a low dose (possibly followed by dose escalation), immediate discontinuation of ICI therapy in the event of severe toxicity and rapid treatment with corticosteroids. History of prior GVHD seems to be an adverse risk factor for subsequent GVHD and both preclinical and clinical data indicate that a shorter interval between allo-HCT and ICI therapy is associated with a higher risk of immunological complications (46, 50–52, 62), which should be taken into consideration before initiation of ICI treatment.

Clinical trials in the solid cancer setting suggested that severe development of severe irAEs was more frequent with ipilimumab compared to nivolumab (30, 31, 85–87). In contrast, frequency and severity of irAEs and GVHD seem to be slightly higher in anti-PD-1 treated patients than in anti-CTLA-4 treated patients in the post allo-HCT setting, although direct evidence from head-to-head comparisons of these two scenarios is lacking. Therefore, on the one hand, differences in study design and patient characteristics, including timing of ICI treatment post allo-HCT, graft source, GVHD prophylaxis, and history of prior GVHD, might be a reason for this discrepancy. On the other hand, the conditioning regimen, GVHD prophylaxis, allogeneic HSC transfer, and increased pro-inflammatory milieu post allo-HCT are important factors that influence the immune system and might account for differences in the ICI toxicity profile. Another issue might be the mechanistic differences between CTLA-4 and PD-1 blockade (12, 88). Since CTLA-4 plays a more important role in early immune responses within lymph nodes and the T cell priming process and PD-1 rather during later phases of the immune response, peripheral T cell activity and maintenance of self-tolerance, toxicity levels may be skewed in favor of CTLA-4-blockade in a context without alloreactivity, that is, solid tumors.

Future studies are required to further delineate the pathophysiological mechanisms and assess the prophylactic and treatment strategies to minimize irAE and GVHD development while preserving the therapeutic efficacy of ICI.

## Author Contributions

NK collected and reviewed literature, discussed the studies, and wrote the original draft of the manuscript. DAR, RK, and RZ contributed to writing and critically revised the manuscript. All authors contributed to the article and approved the submitted version.

## Conflict of Interest

RZ received honoraria from Incyte, Novartis and Mallinckrodt. The remaining authors declare that the research was conducted in the absence of any commercial or financial relationships that could be construed as a potential conflict of interest.
